# Protocol for the precise activation of intradental neurons in mice via electrical stimulation

**DOI:** 10.1016/j.xpro.2026.104386

**Published:** 2026-03-02

**Authors:** Shuhao Wan, Elizabeth A. Ronan, Aditi Jha, Akash R. Gandhi, Brian S.C. Constantinescu, Kevin P. Pipe, Joshua J. Emrick

**Affiliations:** 1 Department of Mechanical Engineering, University of Michigan, Ann Arbor, MI 48109, USA; 2 Department of Biologic and Materials Sciences, University of Michigan, Ann Arbor, MI 48109, USA

**Keywords:** Cell biology, Neuroscience, Systems biology

## Abstract

Intradental neurons encode tooth sensation, yet identifying them without sacrificing tooth structural integrity remains challenging. Here, we present a protocol for the non-invasive electrical stimulation of intradental neurons with single-molar tooth resolution in anesthetized mice. We describe details for head fixation to access molars. We then provide steps to deliver direct current (DC) pulses to an individual molar while simultaneously monitoring induced current. The protocol can be combined with neural imaging to identify and characterize intradental sensory responses and circuits.

For complete details on the use and execution of this protocol, please refer to Ronan et al.^1^

## Highlights

• Fabricating a custom mouse stereotaxic stage and retractors for molar access
• Assembly of an electrical stimulator setup to target individual molars
• Procedures for selective intradental neuron activation in anesthetized mice

## Abstract (editor highlights)

Publisher’s note: Undertaking any experimental protocol requires adherence to local institutional guidelines for laboratory safety and ethics.

## Graphical abstract

**Figure:**
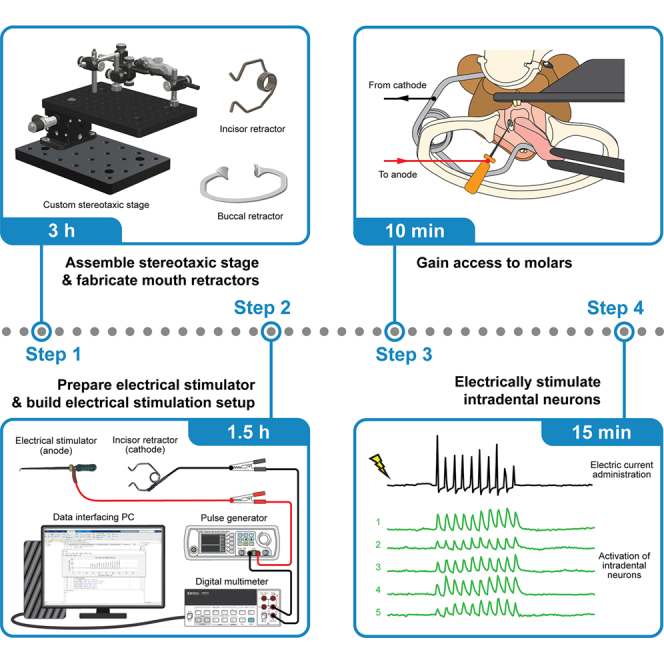

## Before you begin

Tooth sensation and pain are mediated by trigeminal sensory neurons that innervate the tooth’s inner pulp and dentin (intradental neurons).^1^^,^^2^^,^^3^^,^^4^ As projections of intradental neurons terminate beneath the tooth’s hard enamel, non-invasive and precise methods to achieve their activation are technically challenging. This protocol describes a procedure to selectively and repeatably activate intradental neurons using controlled electrical stimulation without inducing tooth damage, as validated by in vivo trigeminal calcium imaging in Ronan et al.^1^ The approach enables targeted neuronal activation while maintaining the structural integrity of the tooth and can also be applied in the context of pulpitis or tooth disease models.

In this protocol, we first describe the preparation of a custom stereotaxic stage and the fabrication of custom incisor and buccal retractors. These steps provide sufficient optical and physical access to the oral cavity and mouse molar teeth in anesthetized, head-fixed mice. Next, we describe how to construct a custom electrical stimulator setup to administer electric pulse to an individual molar. Finally, we provide instructions to combine the use of these approaches to achieve targeted activation of intradental neurons. These procedures provide a flexible method for repeatable activation of intradental neurons without inducing tooth mechanical damage. They are also applicable to functional studies of intradental neurons in the context of pulpitis or diseases compromising tooth integrity.

### Component design and assembly to fabricate a custom stereotaxic stage for head fixation and isoflurane anesthesia

**Timing: 2 h**

This section describes a custom stereotaxic configuration optimized for front-view access to the mouse’s oral cavity. This stereotaxic setup positions the mouse and oral cavity near the front platform edge and incorporates a low-profile, 3D printed palate mounting bar that enables simultaneous skull stabilization and isoflurane delivery. The custom configuration was used in combination with in vivo trigeminal ganglion (TG) calcium imaging,^1^ and includes a goniometer to enable fine tuning of the mouse’s position for subsequent calcium imaging of the neurons at the TG surface.

***Note:*** Any stereotaxic setup that provides stable head fixation and provides sufficient access to the oral cavity and molars can be used.

**Figure 1:**
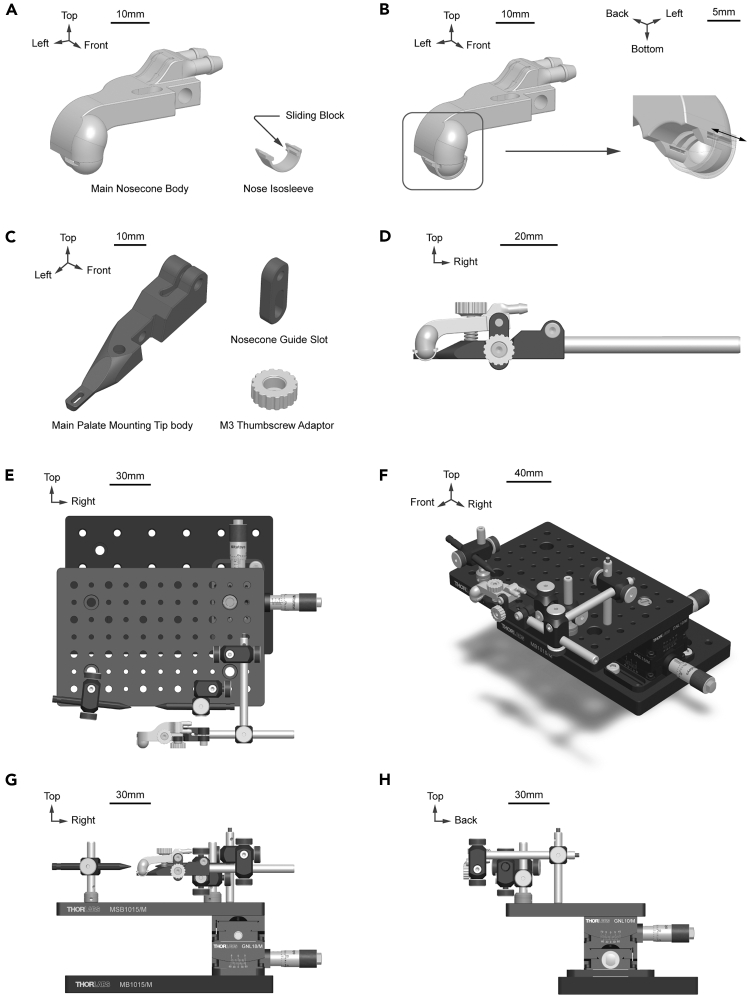
Overview of the custom nosecone and assembled stereotaxic stage for mouse restraint, stabilization, and delivery of isoflurane anesthesia (A and B) CAD image of the custom nosecone that consists of two 3D printable subcomponents, the main nosecone body and the adjustable nose isosleeve. Embedded channels on the main nosecone body are designed for connection to an isoflurane system for inhalation and waste gas expiration. A sliding nose cap (isosleeve) can be adjusted based on animal size to enhance fitting and minimize isoflurane leakage. (A) 3D printable custom nosecone components. Scale bar, 10 mm. See Data S1 and Table S2 for associated STL and CAD files. (B) Schematic of the custom nosecone. Scale bar, 10 mm (left), 5 mm (right). (C) CAD image of the custom palate mounting tip that consists of three 3D printable subcomponents, the main palate mounting tip body, the nosecone guide slot, and the M3 thumbscrew adapter. Scale bar, 10 mm. See Data S1 and Table S2 for associated STL and CAD files. The main palate mounting tip body features an insert slot for the maxillary incisors on one end and a 6mm optical post adapter on the other end. The nosecone guide slot enables height adjustment of the nosecone to accommodate mice with variant sizes. The M3 thumbscrew adapter converts socket head cap M3 screws into thumbscrews to facilitate manual tuning. (D) Schematic of the palate mounting bar assembly. Scale bar, 20 mm. The front thumbscrew can be loosened to release the nosecone guide slot, allowing for nosecone height adjustment to accommodate mice with variant sizes. The top thumbscrew can be loosened while mounting/demounting the mouse and tightened to secure the mouse during procedures. This assembly ensures consistent delivery of anesthesia while stabilizing the animal’s snout. (E–H) Schematics of the custom assembled stereotaxic stage including the nose-cone palate mounting bar. The palate bar is mounted to an optical post and clamp assembly enabling fine-tuning of the angle and height of the mouse’s snout. Additional optical posts and clamps can be used to adjust and secure ear bars enabling skull fixation. The base of the stereotaxic stage assembly consists of two optical breadboards connected via a goniometer. This enables additional adjustments of the head angle following skull stabilization to improve optical access to the mouse’s face and oral cavity during experiments. (E) Top view. Scale bar, 30 mm. (F) Isometric view. Scale bar, 40 mm. (G) Front view. Scale bar, 30 mm. (H) Right view. Scale bar, 30 mm.

***Note:*** The custom nosecone consists of two 3D printable subcomponents, the main nosecone body and the adjustable nose isosleeve. The main nosecone body features embedded channels for isoflurane inhalation and waste gas expiration, and the isosleeve is designed to enhance fitting between the nosecone and the mouse’s snout to minimize isoflurane leakage. Corresponding STL file names are listed in Table S2, and files are provided in Data S1 and available on the Emrick Lab GitHub page (see resource availability). 3D printing materials are listed in Table S3.

***Note:*** We recommend using resin 3D printing techniques such as Stereolithography (**SLA**), Masked Stereolithography (**MSLA**), and Digital Light Processing (**DLP**) to print the custom nosecone components. In the slicing software, we recommend the following setup: orient the main nosecone body front-side-up, orient the isosleeve front-side-down, apply a 5 mm elevation from the build platform, generate supports only from the build platform, and use a 5 μm layer thickness for optimal print quality. Adjust other printer and resin settings accordingly based on the specific printer and resin being used.

1. Acquire necessary commercial components for fabricating the custom stereotaxic stage (Table S1).
2. 3D print the custom nosecone components (Figure 1A; Tables S2 and S3, Data S1).

3. Assemble the custom nosecone (Figure 1B).
  a. Hook the sliding block on one side of isosleeve onto the corresponding groove on the main nosecone body.
  b. Gently push the isosleeve up until both sliding blocks are constrained by the grooves.
    **CRITICAL:** If the sliding blocks are oversized, adjust printing parameters by reducing layer exposure time and accommodating for resin shrinkage and tolerance compensation.
  c. Test the tolerance by sliding the isosleeve forward and backward.
4. 3D print the custom palate mounting tip components (Figure 1C; Tables S3 and S4, Data S1).

***Note:*** The custom palate mounting tip consists of three 3D printable subcomponents, the main palate mounting tip body, the nosecone guide slot, and the M3 thumbscrew adapter. The main palate mounting tip body features a maxillary incisor insert slot on one end and a 6 mm optical post adapter on the other end; the nosecone guide slot enables height adjustment of the nosecone to accommodate mice with variant sizes; the M3 thumbscrew adapter converts socket head cap M3 screws into thumbscrews to facilitate manual tuning.

***Note:*** Filament-based 3D printing techniques such as Fused Deposition Modeling (**FDM**) or Fused Filament Fabrication (**FFF**) are recommended for easier post processing and wider material compatibility.

5. Assemble the custom palate mounting bar (Figure 1D).
  **CRITICAL:** Please refer to the model for details on parts’ spatial arrangement.
  a. Use the soldering iron to insert three M3x4mm knurled nuts into the main palate mounting tip body.
  b. Attach M3 thumbscrew adapters to one M3x12mm and one M3x18mm socket head cap screw.
    ***Note:*** Tight fit between M3 thumbscrew adapter and M3 screw is expected.
    i. Apply super glue to bind the M3 thumbscrew adapters to the screws.
  c. Use one M3x14mm socket head cap screw to attach nosecone guide slot to the custom nosecone. Ensure free rotation between the two components.
    ***Note:*** The hole on the nosecone guide slot is unthreaded and has a diameter smaller than the screw diameter. Apply gentle force to tap the screw through the hole.
  d. Use the M3x12mm thumbscrew prepared in step 5b (Before You Begin) to attach the custom nosecone and nosecone guide slot to the main palate mounting tip body. Tighten the screw.
    ***Optional:*** Place an M3 washer between the thumbscrew and the nosecone guide slot.
  e. Place a 0.4x5x10 mm, 7 laps compression spring between the custom nosecone and the main palate mounting tip body; penetrate the compression spring with the M3x18 mm thumbscrew prepared in step 5b (Before You Begin). Slightly tighten the screw to maintain connection.
    ***Optional:*** Place an M3 washer between the thumbscrew and the custom nosecone.
  f. Insert an MS3R optical post into the main palate mounting tip body.
    i. Use a M3x10mm socket head cap screw to lock the two components.
6. Connect the custom nosecone to the isoflurane delivery system by attaching standard tubing to the inlet and outlet barbs.

**CRITICAL:** Follow the arrow markings on the nosecone to determine the inspiration versus expiration channels. Ensure that tubing connections are securely attached to the nosecone barbs to maintain stable anesthesia during experiments.

7. Assemble the custom stereotaxic stage (Figures 1E–1H).
  a. Connect the top platform to the goniometer using corresponding thread adapters.
  b. Connect the bottom platform to the goniometer using M6x10mm socket head cap screws.
  c. Position and mount the following components on the top platform, referring to Figures 1E–1H):
    i. Optical posts, post clamps, and corresponding thread adapters.
    ii. Stereotaxic ear bars.
    iii. Palate mounting bar assembly.

***Note:*** Adjust the positions of optical posts and stereotaxic stage components as needed based on mouse size.

**CRITICAL:** Ensure all clamps and adapters are securely tightened to prevent motion during experimental procedures. Confirm that the assembled setup provides unobstructed optical and physical access to the oral cavity.

***Note:*** Make sure that the stereotaxic stage is assembled so that the mouse’s mandible is hanging over the edge of the platform to improve physical accessibility to the oral cavity.

### Preparation of custom incisor and buccal retractors

**Timing: 1 h**

This section outlines the fabrication of custom retractors to enable optical and physical access to molars in anesthesized mice. The designed incisor retractor separates the maxillary and mandibular incisors to open the mouth vertically. The buccal retractor moves the lateral buccal mucosa and cheek tissue structures away from midline horizontally to expose the molars.

***Note:*** The incisor retractor is made from a spring wire and serves as the cathode of the electrical stimulation system. The 3D printed buccal retractor is interchangeable with any properly electrically insulated commercial retractor.

**Figure 2:**
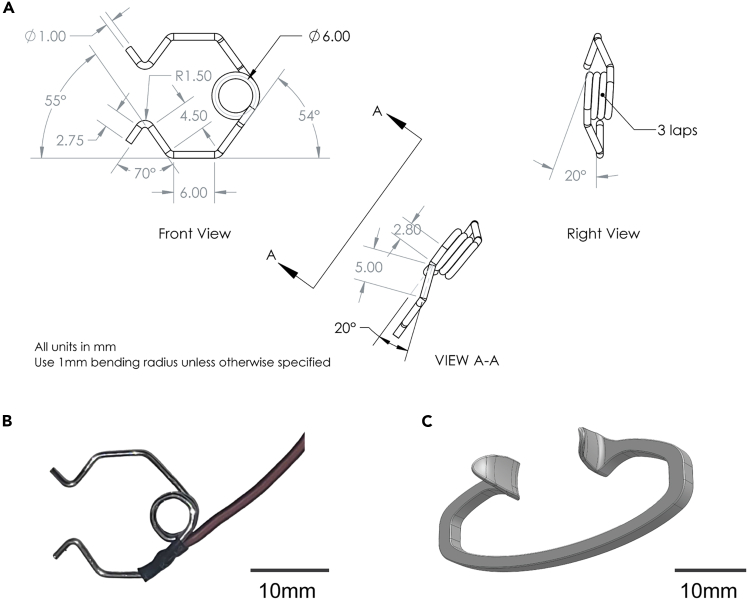
Overview of custom mouth retractors for use with molar electrical stimulation (A and B) (A) Drawings of the incisor retractor with recommended assembly angles for the spring wire. All lengths provided in mm. (B) Snapshot image of the incisor retraction with attached ground wire. Scale bar, 10 mm. (C) CAD image of the 3D printable buccal retractor. See Data S1 and Table S2 for associated STL and CAD files. Scale bar, 10 mm.
9. Fabricate the buccal retractor (Figure 2C; Table S3, Data S1).
  a. 3D print using non-brittle, durable material.

***Note:*** Filament-based 3D printing techniques such as Fused Deposition Modeling (**FDM**) or Fused Filament Fabrication (**FFF**) are recommended for durability and material compatibility.IP

8. Fabricate the grounded incisor retractor by bending a spring wire as depicted (Figures 2A and 2B; Table S5).
  a. Use a pair of pliers to form a 1 mm diameter stainless steel spring wire into the specified configuration (Figures 2A and 2B).
  b. Solder a sufficiently long 22 AWG wire to the incisor retractor.
    **CRITICAL:** Apply stainless steel soldering flux to prevent cold solder joint defects.
  c. Expose 5 mm of the conductor at the opposing end of the wire.
    ***Optional:*** Tin the exposed conductor.
  d. Verify electrical connectivity:
    i. Measure the electrical resistance between the alligator clip and the top and bottom hooks.
      **CRITICAL:** Ensure measured resistance is ≤ 5Ω using a multimeter. Re-solder connections if the measurement exceeds this threshold.

### Preparation of the electrical stimulator

**Timing: 30 min**

This section outlines the fabrication of the electrical stimulator, which serves as the anode to deliver electric potential to an individual molar.

**Figure 3:**
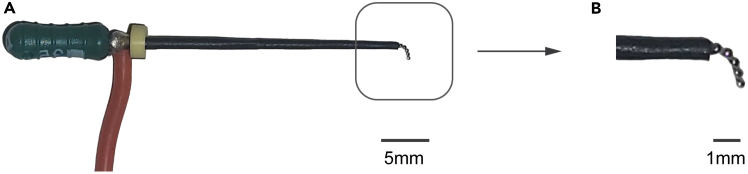
Overview of the stimulator for delivery of electrical potential (A) Snapshot image of the electrical stimulator. Scale bar, 5 mm. (B) Zoomed-in snapshot image of the electrical stimulator tip. Scale bar, 1 mm.

10. Fabricate the electrical stimulator (Figures 3A and 3B; Table S6).
  a. Solder a sufficiently long 22 AWG silicone wire to the metallic region of a 040x31 mm dental file close to its base.
  b. Expose 5 mm of the conductor at the opposing end of the wire.
    ***Optional:*** Tin the exposed conductor.
  c. Verify electrical connectivity:
    i. Measure the electrical resistance between the end of the wire and the electrical stimulator tip.
      **CRITICAL:** Ensure resistance is ≤ 5Ω. Re-solder connections if the measurement exceeds this threshold.
  d. Insulate the metallic region of the dental file, leaving 2 mm of the file tip uninsulated.
    ***Note:*** Alternatively, thin (∼0.2 mm) stainless steel spring wire can replace the dental file described. We recommend using 2:1 ratio miniature heatshrink tubing with an inner diameter <0.4 mm after shrinkage for insulation.
  e. Gently bend the file at 1.5 mm from its tip by ∼70° (Figure 3B).

### Construct the electrical stimulation setup

**Timing: 1 h**

This section provides assembly instructions for the electrical stimulation setup.

11. Assemble the circuit as depicted in the wiring diagram (Figure 4; Table S7).
  a. Attach the BNC male-to-binding post adapter to Channel #1 output of the pulse generator.
  b. Connect the red banana-to-alligator test lead to the anode (red) banana jack of the adapter.
  c. Connect the black banana-to-alligator test lead to the current-in terminal of the digital multimeter (DMM).
  d. Use the black banana-to-banana test lead to connect the cathode (black) banana jack of the BNC male-to-binding post adapter and the current-out terminal of the DMM.
  e. Use an interface cable to connect the DMM to the desktop computer (PC).

**Figure 4:**
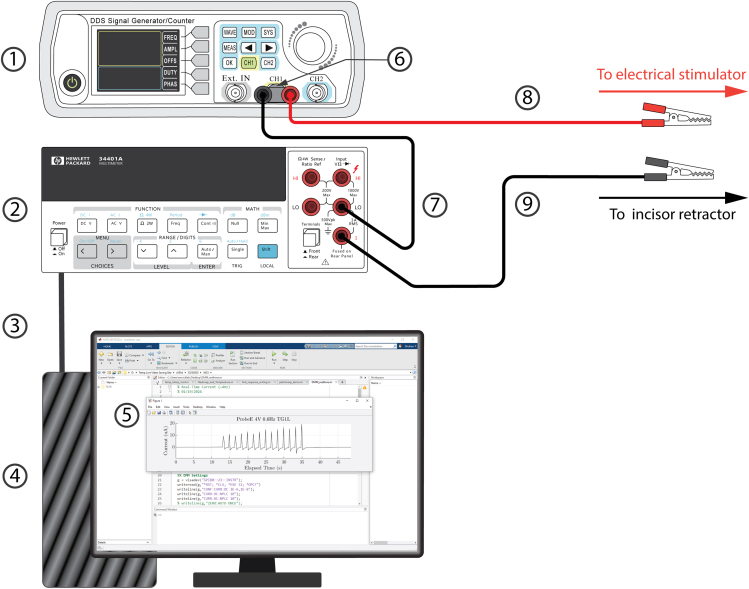
Wiring diagram of the electrical stimulator setup (1) Pulse generator. (2) Benchtop digital multimeter (DMM). (3) Digital multimeter (DMM) to desktop computer interface cable. (4) Desktop computer (PC). (5) Interfacing software to display real-time current readout. (6) BNC male-to-binding post adapter. (7) Black banana-to-banana test lead. (8) Red banana-to-alligator test lead (connect to the electrical stimulator). (9) Black banana-to-alligator test lead (connect to the incisor retraction).
12. Preset the pulse generator.
  a. Follow the user manual to configure and save the pulse generator waveform.

**CRITICAL:** Preset a DC “Pulse” waveform with a 0.6 Hz frequency, 4 Volts amplitude, 2 Volts offset, and 12% duty cycle.

13. Set up DMM and PC communication according to the user manual in the desired interfacing software (for current monitoring).

***Note:*** We recommend using MATLAB or LabView as the interfacing software.

**CRITICAL:** In the interfacing software, configure the DMM to current measurement mode. Set the measurement range to μA. Designate an appropriate measurement resolution such that the acquisition frequency ≥8 Hz.

14. Connect a 1 MΩ resistor (Table S8) between the red and black alligator clips (in series to the circuit). Enable pulse generator Channel #1 output and monitor real-time current readout.

**CRITICAL:** Ensure the peak current readout is 4±0.5 μA.

### Innovation

This approach is inspired by the clinical “electrical pulp test,” which assesses tooth pulp viability based on a patient’s perception of a mild electrical stimulus.^5^^,^^6^ While targeted electrical stimulation of molars has been attempted in vivo in large animal models during electrophysiology recordings,^2^^,^^7^^,^^8^^,^^9^^,^^10^ prior studies lacked precise control over stimulation parameters. Our protocol details the fabrication and use of a custom electrical stimulator that delivers controlled, repeatable electrical pulses to individual molars in mice, a genetically-tractable model, enabling systemic activation of intradental neurons in vivo. Combined with trigeminal calcium imaging, this workflow represents the first application of contemporary neuroscience techniques to monitor and activate intradental neuron activity in real time, providing a platform for mechanistic studies of tooth sensation and pain in health and disease models.

### Institutional permissions

Users of this protocol must obtain required institutional approvals for the use of animals before beginning. All animal experiments described were conducted in accordance with protocols approved by the University of Michigan Institutional Animal Care and Use Committee following NIH guidelines.

## Key resources table

**Table:** 

REAGENT or RESOURCE	SOURCE	IDENTIFIER
**Chemicals, peptides, and recombinant proteins**		

Phosphate-buffered saline (PBS, 1X), sterile-filtered	Thermo Scientific	J61196.AP
Isofluorane	MWI Animal Health	501017

**Deposited data**		

CAD models	N/A	https://doi.org/10.5281/zenodo.18234430
STL files for 3D printing	N/A	https://doi.org/10.5281/zenodo.18234430

**Experimental models: Organisms/strains**		

Mouse: Ai95D (RCL-GCaMP6f)-D (C57BL/6J	JAX (The Jackson Laboratory)	Stock no. 028865; RRID: IMSR_JAX:028865
Mouse: Na_v_1.8-Cre	JAX (The Jackson Laboratory)	Stock no. 036564; RRID: IMSR_JAX:036564

**Software and algorithms**		

MATLAB	MathWorks	https://www.mathworks.com/products/matlab.html

**Other**		

Mini-Series Adapter with External M4 Threads and Internal M3 Threads	Thorlabs	MSA4/M
Mini-Series Adapter with External M6 Threads and Internal M3 Threads	Thorlabs	MSA6/M
Large Dual-Axis Goniometer, 25.4 mm Distance to Point of Rotation	Thorlabs	GNL20/M
Mini-Post Right-Angle Post Clamp Fixed 90° Adapter	Thorlabs	MSRA90
Mini-Series Optical Post Ø6 mm L = 1.5	Thorlabs	MS1.5R
Mini-Post Swivel Post Clamp 360° Continuously Adjustable	Thorlabs	MSWC
100 mm x 150 mm x 9.5 mm Mini-Series Aluminum Breadboard, M4 and M6 High-Density Taps	Thorlabs	MSB1015/M
Aluminum Breadboard, 100 mm x 150 mm x 12.7 mm, M6 Taps	Thorlabs	MB1015/M
M6 x 1.0 Threaded Counterbore Adapter	Thorlabs	CBA6M
Adapter with External M6 x 1.0 Threads and External M4 x 0.7 Threads	Thorlabs	AP6M4M
Adapter with External M4 Threads and External M3 Threads	Thorlabs	AP4M3M
Mini-Series Optical Post, Ø6 mm, L = 2″	Thorlabs	MS2R
Mini-Series Optical Post, Ø6 mm, L = 3″	Thorlabs	MS3R
M6 x 1.0 Stainless Steel Cap Screw, 10 mm Long, 25 Pack	Thorlabs	SH6MS10
Standard Ear Bars and Rubber Tips for Mouse Stereotaxic	Stoelting	51648
Chitu Systems Conjure Rigid Resin with Engineering-Like Features for Functional Gadgets Resin Printing,Low Shrinkage for Articulated Figure LCD 3D Printing (White,1000g)	Amazon	B09JGB3HFT
Bambu PLA-CF (Black)	Bambu Lab	14100
Bambu PLA Basic (Jade White)	Bambu Lab	10100
1110 Pcs M3 Small Metric Screws with Nuts, Hex Socket Head Cap and Nut Assortment Kits, Stainless Steel Replacement Machine Fastener Screws and Bolts Nuts (Silver 1110)	Amazon	B0CMQG542V
Kadrick 520Pcs M2 M3 M4 M5 Threaded Inserts Assortment Kit for 3D Printing Components, Metric Brass Knurled Nuts, Insert by Heat into Plastic Parts	Amazon	B0D5V3TZLB
Dianrui 300PCS Compression Springs Assortment Kit 23 Different Sizes Small Spring 304 Stainless Steel Mechanical Mini Springs for DIY Repair Project	Amazon	B0BVTDP29W
High-Temperature Silicone Rubber Tubing for Air&Water	Mc-MASTER CARR	5293N12
Stainless Steel Spring Wire, Music Wire 15.7 in Length, 0.2–3 mm Diameters, Full Hard	Amazon	B0DNCMQJDN
BNTECHGO 22 Gauge Flexible 2 Conductor Parallel Silicone Wire Spool Red Black High Resistant 200 deg C 600V for Single Color LED Strip Extension Cable Cord, Model,25ft Stranded Tinned Copper Wire	Amazon	B077XBWX8V
Chanzon 2:1 Ratio - 80Ft Roll - 1/42" (0.6mm) Heat Shrink Tubing 2:1(25M Total Length) Black Polyolefin Sleeving Wrap Shrinking 2 to 1 1pcs	Amazon	B0B618H243
SBSK Silver Solder 3/64 1/2 oz STAR2000 by Stay-Brite	Amazon	B0015H6JYS
Edge Hedstroms Hedstrom File 31 mm Size #35 Stainless Steel Green 6/Pk	Henry Schein	4680323
Koolertron DDS Signal Generator Counter, 2.4in Screen Display 15MHz High Precision Dual-channel Arbitray Waveform Generator Frequency Meter - US Plug	Koolertron	GH-CJDS66-A
Agilent/HP 34401A DMM	Keysight Technologies	N/A
Cal Test Electronics CT2410: Adapter Connector BNC Male To Binding Post, Double Black	DigiKey	BKCT2410-ND
B&K Precision TL 5A: 40.0" (1016.00mm) Banana Plug, Single To Alligator Clip, Insulated Patch Cord	DigiKey	TL-5A-ND
12.0" (304.80mm) Banana Plug, Single, Stackable To Banana Plug, Single, Stackable Patch Cord 5000VDC (5kV)	DigiKey	B-12-0
GPIB Interface USB 2.0	DigiKey	2770-154939-01-ND
RES 1M OHM 1% 0.6W AXIAL	DigiKey	LR1F1M0
YIHUA 8786D I 2 in 1 Hot Air Rework and Soldering Iron Station with °F/°C, Cool/Hot Air Conversion, Digital Temperature Correction and Sleep Function	Amazon	B07SCPZJYS
Keiba T-346 Radio Pliers, Multi Type, 5.9 inches (150 mm)	Amazon	B002AW1Y5G
Bambu Lab X1C 3D Printer	Bambu Lab	N/A
Saturn 4 Ultra	ELEGOO	N/A
Absorbent Paperpoints	Meta Dental Corp	1201–204
Polyethylene tubing (.011″ x .024″) per ft., 200 ft	Braintree Scientific	PE10200FT
SomnoSuite® Low-Flow Anesthesia System	Kent Scientific	SS-01
Ophthalmic ointment	Fisher Scientific	NC0490117
Blunt Retractor Tips, 2.5 mm	Fine Science Tools	18200–10

## Materials and equipment

The electrical components (e.g., DMM, pulse generator, cables) used in this protocol can be substituted with any other models with similar functions. The 3D printing materials applied in this protocol (Table S3) can be substituted with either the same materials from other origins, or alternative materials with acceptable mechanical properties (see Table 1).

**Table 1:** Recommended alternative 3D printing materials with acceptable mechanical properties

REAGENT or RESOURCE	SOURCE	IDENTIFIER	NOTE
Siraya Tech Nylon Mecha White Blu Tough LCD Resin (1L)	MatterHackers	M-ZHG-T8DX	For custom nosecone
PETG-CF (Black)	Bambu Lab	31100	For custom palate mounting tip
ABS-GF (Black)	Bambu Lab	41101	For custom palate mounting tip
PA6-CF	Bambu Lab	72100	For custom palate mounting tip
PAHT-CF	Bambu Lab	70100	For custom palate mounting tip
PETG HF (White)	Bambu Lab	33100	For buccal retractor
ABS (White)	Bambu Lab	40100	For buccal retractor

## Step-by-step method details

### Gaining access to mandibular molars using the custom stereotaxic stage and retractors

**Timing: 10 min**

**Figure 5:**
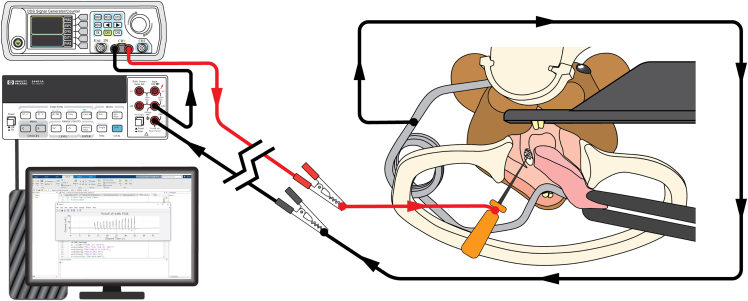
Overview of experimental setup Schematic of the electrical circuit with the mouse in the series. Arrows indicate the current direction.

This section details how to gain optical and physical access to the oral cavity in an anesthetized, head-fixed mouse using the custom stereotaxic stage, grounded incisor retractor, and buccal retractor (Figure 5).

1. Induce anesthesia in the mouse.
  a. If using a Somnosuite Low-Flow Anesthesia System (Kent Scientific) for anesthesia delivery, for an adult mouse (∼8 weeks of age), start isofluorane at 4%–5% for induction.
  b. Confirm adequate plane of anesthesia by monitoring the mouse for loss of righting reflex.
2. Switch anesthesia flow to the nosecone. We recommend beginning at 1.5%–2% at 100 mg/mL using a Somnosuite Low-Flow Anesthesia System (Kent Scientific).
3. Mount mouse on the palate bar.
  a. Monitor and maintain the animal’s body temperature as described in the user’s animal protocol.
  b. Hook the maxillary incisors into the slot on the palate bar.
  c. Gently clamp the nose using the top thumbscrew.
  d. Slide and close the isosleeve against the snout to ensure adequate delivery of isoflurane to maintain a deep plane of anesthesia.

**CRITICAL:** Ensure sufficient anesthesia is achieved by confirming the loss of withdrawal from toe or tail pinch.

**CRITICAL:** Monitor breathing rate to ensure mouse is stable. If gasping occurs, loosen the nose isosleeve and/or adjust anesthesia concentration and/or flow rate.

4. Apply ophthalmic ointment to both eyes to prevent corneal drying.
5. Trim whiskers close to the mouse’s face (∼1 mm).
6. Raise and stabilize the skull with ear bars.
  a. Angle the palate bar so that the head is aligned with the spine in a neutral position.
  b. Insert the first ear bar into the ear canal and gently tighten the screw to prevent sliding.
  c. Insert the contralateral ear bar and tighten the screw.
  d. Gradually raise the ear bars alternately to elevate the skull without raising the mouse from the platform.
  e. Adjust the palate bar height as needed to maintain a flat skull plane while raising the ear bars.
  f. Confirm skull stability by gently applying pressure on the top of the skull; tighten both ear bars and nose-cone screws securely before proceeding.

***Optional:*** For TG calcium imaging, proceed to surgically expose the trigeminal ganglia surface.^1^^,^^11^

7. Insert the incisor retractor to vertically open the mouth.
  a. Place the hooks of the incisor retractor between the upper and lower incisors.
  b. Gently wedge it in place so that it applies even tension to the maxilla and mandible, keeping the mouth open.
8. Place the buccal retractor in the mouth to retract the lateral buccal mucosa away from the gingiva/molars.

***Note:*** Placement of buccal retractor can be readjusted to improve optical and physical access to the mandibular molars.

### Application of electrical stimulation to mandibular molars

**Timing: 15 min**

This section presents the steps to apply molar electrical stimulation after gaining access to mandibular molars.

9. Attach the alligator clip on red banana-to-alligator test lead to the silicone wire soldered on the incisor retractor (Figure 4, component 8).
10. Attach the alligator clip on black banana-to-alligator test lead to the silicone wire soldered on the electrical stimulator (Figure 4, component 9).

**CRITICAL:** For steps 9 and 10 (Step-By-Step Method Details), ensure each alligator clip is only in contact with the exposed conductor of the wire. Attaching the alligator clip to the insulation layer can result in unstable electrical contact.

**CRITICAL:** Ensure the two alligator clips do not contact each other and avoid placing them directly on metallic surfaces to avoid the risk of a short circuit.

11. Position the electrical stimulator to contact the molar. Contact must be maintained throughout the remaining experimental paradigm.

***Optional:*** Use insulated “helping hands” or a 3-axis manipulator to stabilize the electrical stimulator. This facilitates precise alignment and sufficient contact between the file tip and the target molar surface.

**Figure 6:**
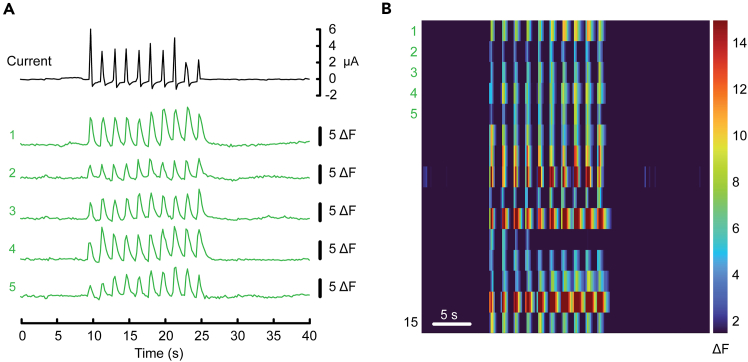
Example application of the protocol combined with trigeminal calcium imaging (A) Example electric current trace (top) and representative traces of GCaMP6f from individual intradental neurons showing merged single frames of Ca^2+^ response (ΔF) (bottom). Data were obtained from an adult *Scn10a-*Cre; Ai95(RCL-GCaMP6f)-D (Ai95D) mouse. (B) Example heatmap from the same TG showing responses of 15 intradental neurons including numbered traces from (A). Scale bar, 5 s. See also Video S1 from Ronan et al., 2025.^1^

12. Initiate current monitoring.
13. Apply the stimulation waveform by enabling pulse generator Channel #1 output.
14. Ensure the peak current readout falls in the 2 to 50 μA interval (Figure 6A).

**CRITICAL:** Enhance electrical conductivity if the current measurement is lower than 1 μA. Lightly moisten a sterile paper point (XC size recommended) with 1X PBS at 20°C (follow manufacturer’s guidance for long-term storage and stability) and gently touch the gingival region or tooth surface near the stimulator tip.

**CRITICAL:** Measured peak current must not exceed 100 μA. Excessively high current could elicit trigeminal neuron activations not specific to the molar and cause irrevocable damage to intradental neurons.

***Note:*** This step helps maintain adequate oral moisture and improves contact between the stimulator and the molar. Reverse-action tweezers are recommended for steady handling of the paper point.

## Expected outcomes

This protocol enables the selective activation of intradental neurons in vivo and has been validated using in vivo calcium imaging of the TG.^1^ Electrical stimulation of an individual mandibular molar reliably evokes calcium responses in approximately 12–22 superficially visible intradental neurons per TG (Example dataset shown in Figures 6A and 6B, also see Figure 1 of Ronan et al.^1^). Following identification of electrically-responsive intradental neurons, this approach enables users to monitor subsequent responses to additional stimuli (i.e., mechanical or thermal). Additionally, this protocol provides a robust framework for assessing functional changes in intradental neuron activity after pulp exposure or following induction of pathological conditions (i.e., pulpitis).

## Quantification and statistical analysis

Electric current output can be exported from interfacing software, e.g., MATLAB. In vivo calcium imaging was analyzed as detailed in Ronan et al., 2025.^1^

## Limitations

We previously demonstrated that each murine molar is innervated by approximately 50 intradental neurons.^12^ However, in our TG calcium imaging approach, we observe responses in an average of ∼16 intradental neurons per TG.^1^ This is a consequence of optical restriction to neurons located near the surface of the ganglion. While we anticipate that electrical stimulation activates most, if not all, intradental neurons within a molar, this has not been directly confirmed.

We recommend using this protocol in conjunction with calcium imaging to directly verify that electrical stimulation successfully activates intradental neurons. Brainstem staining of immediate-early gene expression (e.g., c-Fos/Fos) may also provide validation of activation of neurons proximal to intradental neuron fibers associated with the spinal trigeminal nucleus.^1^ However, because we consistently observe intradental neuron activity when the monitored peak current reaches 2–50 μA, we anticipate that this protocol could also be used to selectively activate intradental neurons in survival experiments where TG calcium imaging is not feasible.

## Troubleshooting

### Problem 1

Isosleeve rail/sliding block and/or screw hole on the 3D printed nosecone exhibit size differences compared to the CAD model (related to steps 2–3 [Before You Begin]). This problem leads to excessively loose or tight fits between mobile components, resulting in assembly failure or unsmooth motion.

### Potential solution

Excessive tolerance in 3D printed components can be induced by: (1) unoptimized tolerance compensation settings during slicing, (2) insufficient part washing during post-processing, and (3) resin shrinkage during curing.

• Optimize the resin shrinkage and tolerance compensation settings in the slicing software. Printing clearance/tolerance test models can assist with fine-tuning these parameters.
• After printing, wash the part for a minimum of 10 minutes to sufficiently remove residual resin. For non-water-washable resins, use fresh isopropyl alcohol (IPA) with a concentration of 95% or higher for part washing.
• If experiencing tight fits, post-process the printed parts by removing excessive material using tools, e.g., files, sandpapers, drill bits, reamers, blades, etc.

### Problem 2

Mouse wakes up from anesthesia during the procedure (related to steps 1–6 [Step-By-Step Method Details]).

### Potential solution

• Confirm animal’s depth of anesthesia is sufficient before proceeding with the procedure. Use the loss-of-toe/tail pinch response to confirm a deep plane of anesthesia is achieved. Monitor continuously throughout the procedure to prevent unexpected arousal during stimulation.
• Adjust isoflurane level.
• Ensure the nosecone is fully sealed around the snout.

### Problem 3

Mouse shows labored or difficult breathing (related to steps 1–6 [Step-By-Step Method Details]).

### Potential solution

Respiratory distress may result from anesthesia level, improper ear bar positioning, or excessive nosecone pressure.

• Lower isoflurane level.
• Reposition the ear bars. Ensure ear bars are secure to stabilize the skull but not compress blood flow.
• Confirm nosecone fit. Make sure the nosecone is not overly tightened around the snout (may occur in larger-than-average mice).

### Problem 4

Following construction of the electrical system set up, when performing the test trial with the 1 MΩ resistor, the interfacing software does not return expected 4 μA peak current readouts (related to step 14 [Before You Begin]).

### Potential solution

There are many possible causes of this problem, including: (1) communication error between PC and DMM, (2) incorrect experimental setup, and (3) mismatch between electrical pulses and data acquisition. To start troubleshooting, increase the pulse generator duty cycle to 99.9% to mimic a static 4 VDC voltage. Enable pulse generator Channel #1 output.

If the interfacing software does not output real-time current readout in μA range.

• Vary the configuration settings (e.g., measurement type) in the interfacing software and ensure the DMM responds correctly to the changes. If the multimeter is unresponsive, refer to the DMM’s user manual to troubleshoot for PC-DMM communication.
• If the DMM is connected via a serial port on the PC, ensure that there is no other software occupying the port.

Real-time updating current readout in μA range indicates that the PC-DMM communication issue has been resolved.

If current readout is oscillating around 0 μA at high frequency, there potentially exists an open circuit in the system and the DMM is picking up background noise.

• Verify all wires are correctly connected (see diagrams in Figures 4 and 5). Pay special attention that in step 11c and 11d [Before You Begin], cables are connected to the multimeter’s current-in/-out terminals.
• Ensure the two alligator clips are in good electrical contact with the 1 MΩ resistor.
• Confirm the pulse generator is outputting via the correct channel (Channel #1).

If current readout is extremely high (≥100 μA), there potentially exists a short circuit in the system.

• Verify all wires are correctly connected (see diagrams in Figures 4 and 5).
• Ensure the two alligator clips are not physically contacting each other.

Steady current readout oscillating around 4 μA indicates a proper electrical stimulation.

Reduce the pulse generator duty cycle to 12%. If the current readout fails to capture all spikes synchronized with the pulse input.

• Verify the DMM’s actual acquisition frequency is no lower than 5 Hz. If so, reduce the measurement resolution to compensate for acquisition frequency.

### Problem 5

During tooth electrical stimulation, the current reading is too low (<1 μA), or no synchronized current pulses are observed (related to steps 12–14 [Step-By-Step Method Details]).

### Potential solution

The electrical signal may not sufficiently reach the molar due to poor stimulator contact with the tooth, or inadequate conductivity at the stimulator-molar interface.

• Verify stimulator placement to ensure the file tip makes stable contact with the molar occlusal surface. Ensure the molar surface is clean and free of food debris or blood. This can interfere with conduction.
• Additional metal components outside of the ones described in the protocol should be insulated to prevent current leakage.
• Improve conductivity by applying a small amount of 1X PBS to the occlusal surface to enhance charge transfer.

## Resource availability

### Lead contact

Further information and requests for resources and reagents should be directed to and will be fulfilled by the lead contact, Joshua J. Emrick (jjemrick@umich.edu).

### Technical contact

Technical questions on executing this protocol should be directed to and will be answered by the technical contacts, Shuhao Wan (shuhwan@umich.edu) and Elizabeth A. Ronan (lizronan@umich.edu).

### Materials availability

Materials used in this protocol are listed in the key resources table. CAD designs and STL files for 3D printing reported in this protocol are accessible on the Emrick Lab GitHub page (https://doi.org/10.5281/zenodo.18234430) and publicly available as of the date of publication.

### Data and code availability

All data reported in this paper will be shared by the lead contact upon request.

## Acknowledgments

This work was supported by NIH grants K22 DE029779 and R01 DE032345 (to J.J.E.), and T32 DE007057 and T32 DC00011 (to E.A.R.).

## Author contributions

All authors gave approval of the final version of the manuscript. S.W., E.A.R., and J.J.E. conceptualized and developed this protocol with assistance from A.J., A.R.G., B.S.C.C., and K.P.P. E.A.R. and S.W. performed electrical stimulation coupled with calcium imaging experiments. S.W., E.A.R., A.J., and J.J.E. drafted and finalized the manuscript with input from B.S.C.C., A.R.G., and K.P.P.

## Declaration of interests

The authors declare no competing interests.

## Supplemental information

Table S1. List of components for fabricating the custom stereotaxic stage (excluding palate bar mounting components), related to Step 1

Table S2. Associated files for 3D printing, related to Steps 2, 4, and 9

Table S3. List of 3D printing materials, related to Step 2, 4, and 9

Table S4. Custom palate mounting tip components, related to Step 4

Table S5. Incisor retractor components, related to Step 8

Table S6. Electrical stimulator components, related to Step 10

Table S7. Wiring components, related to Step 11

Table S8. Resistor, related to Step 14

Data S1. STL files for 3D printing and relevant CAD files, related to Step 1, 2, 4, and 9
